# Supplementary material for: Aptamer based proteomic pilot study reveals a urine signature indicative of pediatric urinary tract infections
Source: PLoS One. 2020 Jul 6;15(7):e0235328. doi: 10.1371/journal.pone.0235328 (PMC7337308; doi:10.1371/journal.pone.0235328)
Supplement: S1 Material — (PDF) [file pone.0235328.s001.pdf]

Supplemental material S1

Primer Sequences:

GUSB:

- Forward: 5’ – ACTGAACAGTCACCGAC – 3’
- Reverse: 5’- AAACATTGTGACTTGGCTAC – 3’

BCL6:

- Forward: 5’ – ATTGTGAGAAGTGTAACCTG – 3’
- Reverse: 5’ – TTTGGGTAGATTCTGAGAAGG – 3’

CXCL1:

- Forward: 5’ – ATGCTAGAACAGTGACAAATC – 3’
- Reverse: 5’ – TCTTCTGTTCCTATAAGGGC – 3’

HSPA1A:

- Forward: 5’ – AATTTCCTGTGTTTGCAATG – 3’
- Reverse: 5’ – AAAATGGCCTGAGTTAAGTG – 3’

MAPK9:

- Forward: 5’ – TCAGATGCAGCAGTAAGTAG – 3’
- Reverse: 5’ – AGGTGAGAGTTCCTTCAATG – 3’

CTSS:

- Forward: 5’- TCTACAGAAGTGGTGTCTAC – 3’
- Reverse: 5’ – CCTTTATTTCTTGCCATCCG – 3’

MMP1:

- Forward: 5’ – AAAGGGAATAAGTACTGGGC – 3’
- Reverse: 5’ – CAGTGTTTTCCTCAGAAAGAG - 3’

CXCL13:

- Forward: 5’ – CATAGTCTGGAAGAAGAACAAG – 3’
- Reverse: 5’ – AAGAATGCAGGTGTTCTTAG – 3’

TAGLN2:

- Forward: 5’ – CACTGACATCTTCCAAACTG – 3’
- Reverse: 5’ – CCTTGGATTTCTTAGGGAAC – 3’

HPV18-E7:

- Forward: 5’ – TGCATGGACCTAAGGCAA – 3’
- Reverse: 5’ – GCTGGGATGCACACCA – 3’

HIST2H2A:

- Forward: 5’ – TTTCCTTGACTCGGAAATG – 3’
- Reverse: 5’ - TCCGAATAGTTGCCCTTG – 3’

CXCL6:

- Waiting on Order
