# Supplementary material for: Aptamer based proteomic pilot study reveals a urine signature indicative of pediatric urinary tract infections
Source: PLoS One. 2020 Jul 6;15(7):e0235328. doi: 10.1371/journal.pone.0235328 (PMC7337308; doi:10.1371/journal.pone.0235328)
Supplement: S2 Material — The support vector machine (SVM) models are trained and selected in an inner leave-one-out cross-validation loop, which involves model-based feature selection and SVM hyperparameter optimization. An outer 5x cross-validation loop evaluates the generalized performance of the optimal model selected from the inner loop on the test set. (PDF) [file pone.0235328.s002.pdf]

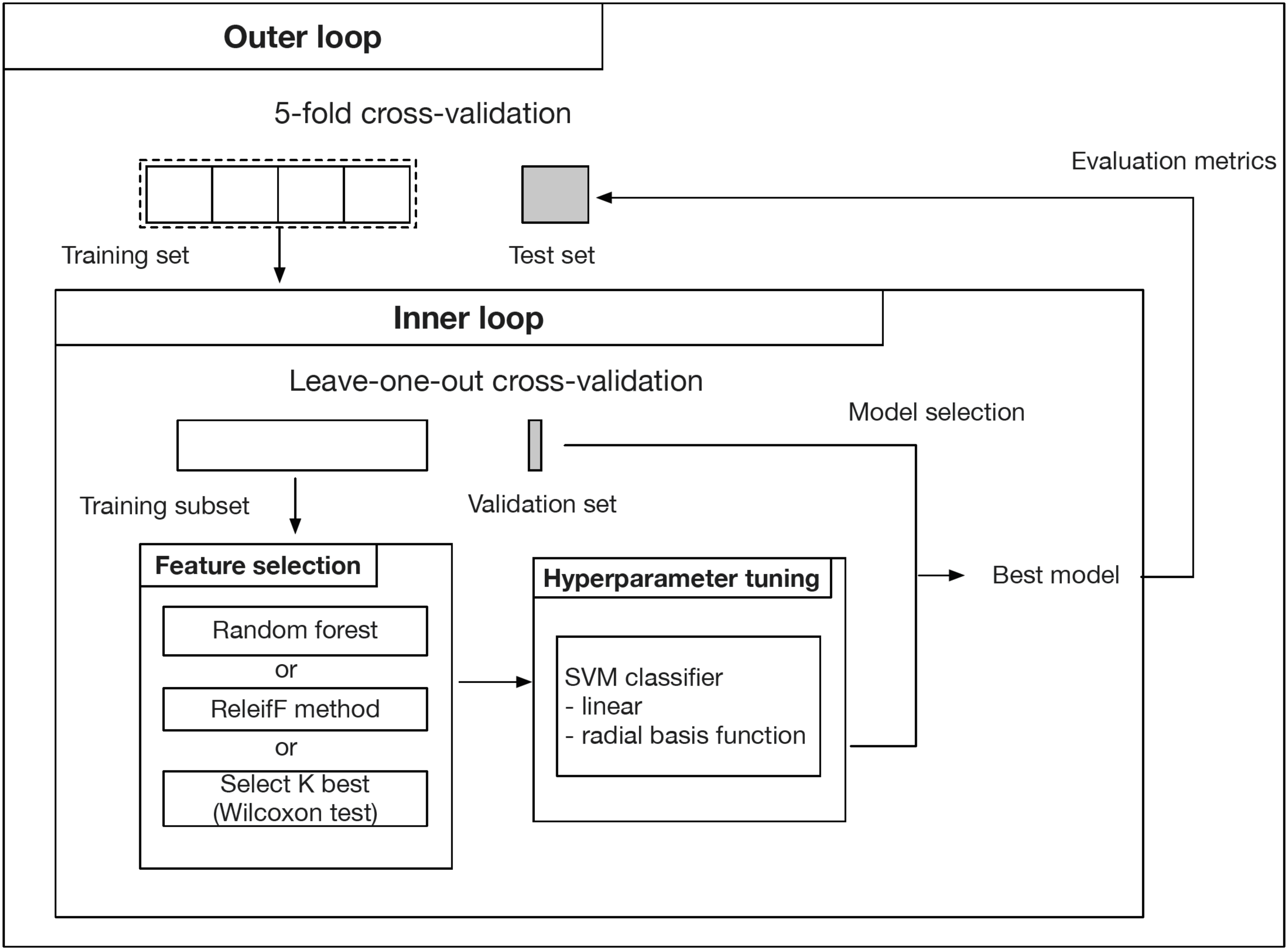

Outer loop
5-fold cross-validation
Training set
Test set
Evaluation metrics
Inner loop
Leave-one-out cross-validation
Model selection
Training subset
Validation set
Feature selection
Random forest
or
ReleifF method
or
Select K best
(Wilcoxon test)
Hyperparameter tuning
SVM classifier
- linear
- radial basis function
Best model
