# Supplementary figures and images for: Aptamer based proteomic pilot study reveals a urine signature indicative of pediatric urinary tract infections
Source: PLoS One. 2020 Jul 6;15(7):e0235328. doi: 10.1371/journal.pone.0235328 (PMC7337308; doi:10.1371/journal.pone.0235328)

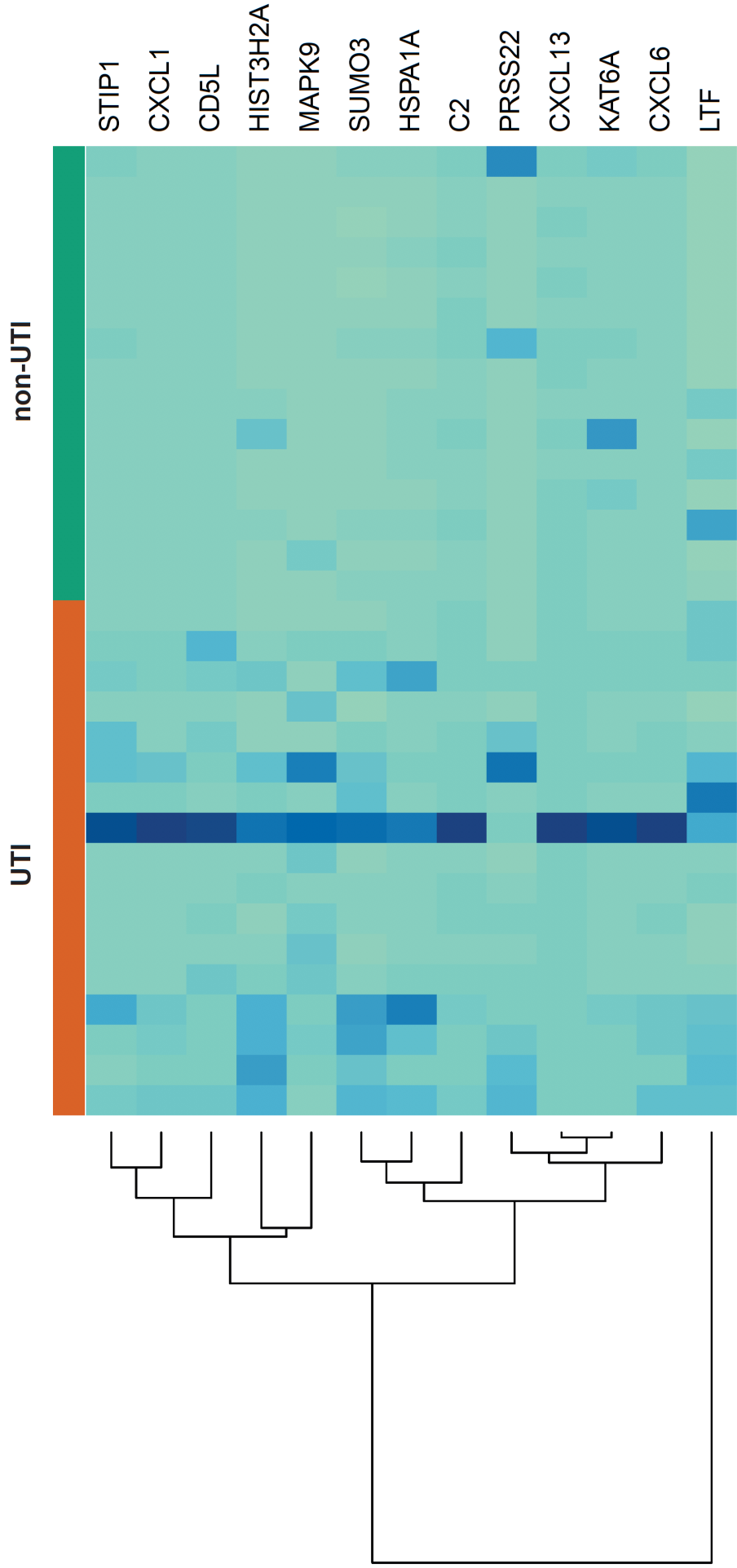

Supplement: S4 Material — (PDF) [file pone.0235328.s004.pdf]
