# Supplementary material for: Aptamer based proteomic pilot study reveals a urine signature indicative of pediatric urinary tract infections
Source: PLoS One. 2020 Jul 6;15(7):e0235328. doi: 10.1371/journal.pone.0235328 (PMC7337308; doi:10.1371/journal.pone.0235328)
Supplement: Attachment — Submitted filename: PLOSone response to reviewers-v1[1].docx [file pone.0235328.s006.docx]

Since the use of dipstix and esterase monitoring was so heavily criticized and it is the current standard it would have been useful to include data regarding the results from the assay to compare performance. The manuscript is comparing a very expensive assay to a relatively inexpensive one. Clearly the intent would be to hone the panel down to a much smaller set of analytes but the comparison might have further strengthened the authors case.

**Response: For this study we purposely chose 16 patients with a positive culture and positive leukocyte esterase (LE) on dipstick, 8 with a negative culture and positive LE and 8 with negative LE and culture. Therefore LE was predetermined to have a sensitivity of 50% and specificity of 33%. In our previously reported larger cohort of 199 patients that we selected our samples from LE >/= small had a sensitivity of 83% and specificity of 85% (PMID:** [**26885759**](https://www.ncbi.nlm.nih.gov/pubmed/26885759)**). We have added this language and the reference to our discussion.**

I question the value/point of providing the three sets of machine learning results as they are quite disparate and there is no significant discussion of the relative merits or limitations of these approaches. Thus it is unclear what this data adds to the current document.

**Response: We have removed the former figure 3 with three machine learning data sets and focused the results on the Random Forest algorithm.**

I found the reporting of fluorescence intensity rather confusing as I am familiar with significantly higher signals with somascan than were listed. This may arise from the normalization to creatinine levels. It would be beneficial to determine if the creatinine levels between each group were significantly different. The data to do this is available in the supplementary tables.

**The urine creatinine levels were not different between groups, P = 0.523 using the Kruskal-Wallis test because the data was not parametric. Additionally we, in the initial submission, presented the ratio of urine biomarker to creatinine with the Somalogic results as relative fluorescent units (RFU) per ml and the creatinine as mg/dl for a dimensionally complex ratio of biomaker(RFU/ml)/ creatine (mg/dl). The higher creatinine volume (dl as opposed to ml) results in lower ratio values. We have revised Figure 2 and Table 2 with the creatinine results as mg/ml so mls cancel out and the results are the more traditional and simple biomarker (RFU)/creatinine (mg) units.**

Response:

I failed to see the value of the inclusion of supplementary figures 6 and 7 as this could just as easily been stated in the discussion. The data derives from protein atlas and has no relationship to the current study other than saying there is evidence to suggest the presence of the candidate proteins in renal and bladder tissues. Similarly I do not see the need/utility of the PCR data on unrelated and incompletely rationalized samples from different tissue sources.

**Response: We have removed supplementary figures 6 and 7 and instead added the presence of candidate biomarkers in renal and bladder tissue to the discussion as recommended.**

Somascan is very robust and sensitive with a very board dynamic range. It is essential to have some form of validation using an antibody based approach to demonstrate that ELISA is actually feasible for these analytes at the concentrations present in urine.

**Response: We have validated the Somalogic results for GM-CSF with an antibody based approach in a separate patient cohort (an adult patient cohort) which resulted in a similar area under the curve value. The results are presented in a new Figure 3. We have also updated the methods, results and discussion based on this finding.**


Comment 1: Language at times is bit difficult to understand, particularly in the paragraph 2-5 of the discussion, the linkage between the paragraphs are also missing.

**Response: We have revised the wording and added linkage for paragraphs 2-5**

Comment 2: Similarly, in the introduction many lines are lengthy which can be difficult to comprehend for readers, should be broken into smaller sentences.
For instance, consider this line:
“Infectious conditions including renal tuberculosis, herpes simplex virus and chlamydia along with noninfectious inflammatory conditions including Kawasaki disease, appendicitis, foreign bodies and interstitial nephritis can cause what has been historically described as sterile pyuria”

**Response: We have shortened sentences and simplified the introduction as recommended.**

Comment 3: Proper referencing is missing at times for instance, consider
“Several challenges have limited protein-based biomarker discovery including the difficulty to develop high throughput assays such as enzyme-linked immunosorbent assay (ELISA) for identifying biomarker candidates.”

**Response: We have add references for the example cited and in other places as well.**

Comment 4: The problems with existing technologies, like ELISA should be briefly discussed in the introduction and how aptamer-based technologies can alleviate them should also be discussed in brief with proper references.

**Response: We have provided an extended explanation for ELISA limitation in the introduction: “Several challenges have limited protein-based biomarker discovery with traditional antibody based ELISAs. Specifically, ELISAs are time consuming to perform and the required antibodies have inherent costs, instability, batch to batch variation, storage requirements limited dynamic ranges and are difficult to multiplex [10-12].”**

Comment 5: It would be good to include some information on the current potential and market of aptamers in Introduction. Some the recent publications on this aspect is as follows.
1- Aptamer-based point-of-care diagnostic platforms
2- Aptamers in the Therapeutics and Diagnostics Pipelines
3- The point behind translation of aptamers for point of care diagnostics
4- Defining Target Product Profiles (TPPs) for Aptamer-Based Diagnostics

**Response: We have added more information about the current and market potential of aptamers in the introduction: Aptamers are being explored as  affordable, sensitive, specific, user friendly point of care tests on a variety of platforms [15]. Aptamers have the ability to perform in formats where antibodies often perform poorly such as homogenous multiplex assays, do not degrade when stored at room temperature as a dry lyophilized reagent at room temperature and have minimal to no batch to batch variation [12]. Thus there is speculation that aptamers may replace antibodies in future diagnostics [16]”**
